# Developing Effective Health Interventions for Women Who Inject Drugs: Key Areas and Recommendations for Program Development and Policy

**DOI:** 10.1155/2012/269123

**Published:** 2012-10-16

**Authors:** Sophie Pinkham, Claudia Stoicescu, Bronwyn Myers

**Affiliations:** ^1^Department of Slavic Languages, Columbia University, 1130 Amsterdam Avenue, Mail Code 2839, New York, NY 10027, USA; ^2^Public Health Policy, Harm Reduction International, Unit 2D12 South Bank Technopark, 90 London Road, London SE1 6LN, UK; ^3^Department of Social Policy and Intervention, University of Oxford, Barnett House, 32 Wellington Square, Oxford OX1 2ER, UK; ^4^Alcohol and Drug Abuse Research Unit, South African Medical Research Council, P.O. Box 19070, Tygerberg 7505, South Africa; ^5^Department of Psychiatry and Mental Health, University of Cape Town, Faculty of Health Sciences Private Bag, Observatory, Cape Town 7935, South Africa

## Abstract

Women who inject drugs face multiple gender-specific health risks and barriers to healthcare access. These gendered factors may contribute to elevated rates of HIV for this population. Though few countries systematically collect gender-disaggregated data related to injecting drug use, evidence indicates that there are large populations of women who inject drugs and who are in need of improved health services, including HIV prevention. Research on the effectiveness of interventions specifically tailored for women who inject drugs, along with the experience of programs working with this subpopulation, suggests that HIV risk practices need to be addressed within the larger context of women's lives. Multifaceted interventions that address relationship dynamics, housing, employment, and the needs of children may have more success in reducing risky practices than interventions that focus exclusively on injecting practices and condom use. Improved sexual and reproductive healthcare for women who use drugs is an area in need of development and should be better integrated into basic harm reduction programs.

## 1. Introduction

There are significant differences in the health status and risk practices of female injecting drug users (IDUs) as compared to male IDU. A recent comprehensive review by the Reference Group to the United Nations (UN) on HIV and injecting drug use found that compared to their male counterparts, women who inject drugs experience significantly higher mortality rates, increased likelihood of injection-related problems, faster progression from first drug use to dependence, higher levels of risky injection and/or sexual risk behaviors, and higher rates of HIV [1]. Similarly, a systematic review of studies from 14 countries found a significantly higher prevalence of HIV among female IDU than among male IDU in settings with high HIV prevalence [2], and a review of studies in nine European Union countries found that the average HIV prevalence was more than 50% higher among female IDU than among their male counterparts [3].

Research on women who inject drugs comes mainly from North America, Western Europe, and Australia. Although there is a growing body of research on female IDU in low- and middle-income countries, to date there has been no systematic analysis of the prevalence of injecting drug use among women internationally, and data on women as a percentage of people who inject drugs is sparse. In the global data holdings on injection drug use and HIV maintained by the Reference Group to the UN on HIV and injection drug use, none of the countries that report injecting drug use have data disaggregated by gender. The Reference Group's global data holdings also show that countries that provide HIV prevention, treatment, care, and support services for people who inject drugs generally fail to disaggregate data based on gender. This makes it difficult to assess disparities in access to services or the degree to which available services respond effectively to women's needs. This is cause for concern, as estimates suggest that women are a sizable minority of people who inject drugs in several settings with large populations of IDU, including Russia, China, and Ukraine (see Table 1).

Existing evidence on the experiences of women IDU globally suggests that in many settings this population faces limited access to some health services, including reproductive health care, prevention of mother-to-child transmission of HIV (PMTCT), and drug treatment [1, 10, 11]. In some settings, particularly in culturally conservative regions such as Central Asia or the Caucasus, the social stigma attached to women's injecting drug use and to HIV and sexually transmitted infections (STIs) can be a formidable barrier to access to harm reduction services such as needle and syringe programs (NSPs), opioid substitution treatment (OST), HIV treatment, sexual and reproductive health care, and other medical services (see Text Box 1). For example, women IDU in Central Asia have reported being afraid to visit a service site because neighbors might see them there and thus find out that they use drugs or are HIV-positive, which could have devastating social consequences [10, 11].

Because women are a minority of the IDU population, they are not always included in relevant health programs. In some countries, for example, antiretroviral treatment (ART) and OST are available in men's prisons, but not in women's [10, 12, 13]. Many harm reduction programs do not respond to the specific needs of women, such as reproductive health care, and provide only a basic package of injecting supplies, condoms, and gender-neutral health information [11]. At the same time, women who use drugs may be excluded from women's shelters and other special services for at-risk women [5, 11, 13] or have reduced access to PMTCT because of their status as drug users [14]. In summary, women who use drugs are often forgotten by services for drug users due to their sex and neglected by services for high-risk women and people with HIV due to their drug use.

Drawing on existing research, this article will analyze key areas in which the experience of female IDU differs from that of male IDU. It will then discuss potential strategies to help better address these areas in HIV prevention services.

### 1.1. Injecting Drug Use and Sexual Relationships

Multiple studies have found that women who inject drugs have greater overlap between sexual and injection social networks than men do, and that they are more likely than their male counterparts to have a sexual partner who injects drugs [1, 5]. This may reflect the greater social isolation of women IDU due to the particular stigma of women's drug use, as well as the fact that women are a minority of the IDU population [15, 16]. Overlap of sexual and injection networks may increase women's risk of acquiring HIV through sexual transmission as well as through unsafe drug injecting. For example, a woman who does not herself share injecting equipment could be exposed to HIV by a sexual partner who does. Some couples share injecting equipment as a gesture of trust or intimacy [17]. Female IDU are more likely than male IDU to be dependent on a sexual partner for help acquiring drugs and injecting [1]. Being injected by someone else has been found to be an independent predictor of HIV incident infection, meaning that dependence increases women's HIV risk [18].

Relationship dynamics can make it difficult for women to access harm reduction services, enter and complete drug treatment (if desired), and practice safer drug use and safer sex [1]. Partners may forbid women to visit health services out of jealousy, or due to social stigma [13]. If a woman wishes to enter drug treatment but her partner does not, he may oppose her decision. Even if he is not opposed, his continued drug use may make it difficult for her to stop or reduce her own.

Intimate partner violence (IPV) is more commonly reported among women who use drugs than among women in the general population. Some studies have estimated that the prevalence of physical and sexual IPV is three to five times higher among women who use drugs as compared to community-based samples of nondrug-using women [1, 19, 20]. (Some of the research discussed here applies to the broader category of women who use drugs, rather than being specific to women IDU. Given that women IDU are a subcategory of women who use drugs, often experiencing severe levels of drug dependence and greater health risks and social marginalization, it is reasonable to assume that women IDU share many of the problems faced by the larger group of women who use drugs.) Problematic drug use among women is often associated with a history of sexual and physical abuse [20–23]. (EMCDDA defines “problem” drug use as “injecting drug use or long duration or regular use of opioids, cocaine and/or amphetamines.” Definitions of “problem,” “hard,” or “heavy” drug use can vary, but generally fit this basic description.) Some evidence suggests that women may be more likely to engage in substance use as a way of self-medicating for mental health issues, such as depression, anxiety, and posttraumatic stress disorder, that are often the result of trauma, abuse, and violence [24–26].Because it can impair a woman's freedom of choice [27, 28], contribute to self-destructive behaviors [29, 30], and cause instability in a woman's living situation and further dependence on her partner [31], IPV can reduce a woman's ability to practice safer sex and safer drug-injecting practices [20, 31].Drug use and IPV often co-occur as part of a cyclical pattern in which the stress and trauma caused by IPV contributes to women's continued drug use, and the activities and behaviors associated with drug use further increase the risk for IPV [32]. A history of violence can also be an obstacle to healthcare access. For example, it may make women feel uncomfortable in a support group, where the majority of participants are men, or when receiving pelvic exams [33]. Where a history of trauma contributes to problem drug use or risky behaviors, it is important that harm reduction and drug treatment programs take this into account, and that staff are trained in how to address these issues appropriately [34].

Although evidence in this area is particularly limited, some research indicates that a significant portion of women IDU have sex with women, and that there is a high prevalence of risky sexual and drug use practices among IDU women who have sex with women (WSW). The stigma and discrimination faced by WSW may contribute to increased levels of drug use, and many WSW have sex with men as well as women, sometimes in transactional situations [35–38].

### 1.2. Injecting Drug Use and Sex Work

There is significant overlap between women's injecting drug use and engagement in sex work (the performance of sexual services for a payment negotiated in advance), especially sex work that takes place on the street (rather than through a brothel which usually offers some measure of physical security). Participation in sex work has been associated with syringe sharing and inconsistent condom use, as well as other risks posed by the dangerous circumstances in which sex work often takes place [1]. Sex workers often risk losing work if their clients or employers find out that they inject drugs, which can deter sex workers from seeking harm reduction services when needed [11]. Sex workers who trade sex for drugs or who work to support a drug habit often work in higher risk situations (e.g., on a highway, where they are alone and very vulnerable to violence, including rape) and may be less likely to use condoms, in part because the pain of drug withdrawal presents a more immediate threat than HIV or STIs [39]. Research has shown that women IDU who engage in sex work have higher HIV prevalence than their IDU peers who do not sell sex [40]. Punitive policy and legal environments often prevent this population from accessing essential HIV prevention services, and exacerbate the vulnerabilities and risks that they face [41].

### 1.3. Women and Drug Treatment

As compared to men, there appear to be a number of differences in women's motivations to enter and complete OST and other types of drug treatment and in the personal dynamics that play a part in treatment success [1]. Many women cite pregnancy as a central reason for treatment entry, though punitive policies that separate drug-using women from their children can deter pregnant women and mothers from entering drug treatment [1]. A partner's entry into treatment is another key factor that can facilitate treatment entry for women [1]. OST and certain other types of drug treatment have been found to be especially effective in helping women reduce their drug use, while detoxification alone is significantly less successful for female IDU than for their male counterparts [1].

### 1.4. Sexual and Reproductive Health and Pregnancy

While harm reduction programs usually include condom distribution and HIV/STI testing (and sometimes treatment), many do not address other aspects of sexual and reproductive health—despite the fact that many women IDU experience unplanned pregnancies [10, 11, 13, 42]. Some women do not realize they are pregnant until relatively late, making it more difficult for them to access appropriate prenatal care, drug treatment (if desired), and other support, or to terminate their pregnancies safely if they so choose [10, 11, 13, 43].

Faced with high levels of stigma and discrimination, often combined with poverty, unstable housing, and other problems, women who inject drugs often have reduced access to prenatal care [10, 11, 13]. In some cases, laws and healthcare practices are directly responsible for this reduced access. In some parts of the United States, laws that criminalize drug use during pregnancy are powerful disincentives for women to get prenatal care and speak openly with their doctors about the best course of treatment, including drug treatment options [44, 45]. In Russia and Ukraine, drug addiction in itself is statutory grounds for abortion and termination of parental rights—this can apply even when a woman has begun drug treatment, since she is still listed on the government registry of drug addicts [46, 47]. Pregnant women who use drugs (including women with HIV) have reported heavy pressure from doctors to have abortions, sometimes late in the pregnancy; doctors have been reported to tell reluctant women that their baby is certain to have severe birth defects, such as missing limbs [46–48]. Doctors also pressure women to give up custody of their newborns and humiliate and intimidate them; this is an obvious reason for women to avoid healthcare during pregnancy [46, 47]. Reduced access to prenatal care can lead to reduced levels of PMTCT among women IDU living with HIV. A 10-year study in Western and Central Europe of ART during pregnancy found that a history of injecting drug use was associated with the risk of not receiving ART and with being diagnosed with HIV late in pregnancy [14].

The World Health Organization (WHO), United Nations Office on Drugs and Crime (UNODC), and the Joint United Nations Program on HIV/AIDS (UNAIDS) comprehensive harm reduction package for the prevention, treatment, and care of HIV among people who use drugs does not include contraceptive methods (other than condoms), pregnancy tests, pre- and postnatal care; or links between harm reduction, drug treatment, and prevention of vertical transmission of HIV [49]. Adding these to the package could help women who inject drugs to better manage their sexual and reproductive health, thus preventing unplanned pregnancies and improving pregnancy outcomes, including through improved access to prevention of vertical transmission of HIV.

Many pregnant women who are dependent on opiates may wish to begin OST or other forms of drug treatment. Prompt, easy access to these services is essential in improving outcomes for women and their children. While there has been some scale-up of OST worldwide [5], information and protocols on OST provision during pregnancy and postpartum (including during stays in maternity hospitals) do not exist in many countries, and OST is sometimes entirely unavailable for pregnant women or women in delivery, whether because doctors believe it is unsafe, or because of regulatory obstacles [10, 11, 13, 34, 46, 47]. This risks treatment interruptions and makes it more difficult for women to access the “treatment of choice” during pregnancy [34]. Long waits in some countries to enter OST and other drug treatment programs, and the complete unavailability of OST in some countries (notably Russia), threaten the health of all IDUs and are especially troubling in the case of pregnant women.

### 1.5. Women, Injecting Drug Use, and Prisons

An increasing number of women worldwide are being incarcerated for drug-related offences; many of these women are drug users in need of healthcare [50–55]. A recent study found that more than one in four female prisoners in Europe and Central Asia had been convicted of a drug offence [56]. The number of women incarcerated for drug-related offences in Russia is more than double the total number of female prisoners in all EU countries combined [56]. In Tajikistan, up to 70% of all female prisoners have been incarcerated for drug-related crimes [56]. The dual criminalization of sex work and drug possession puts sex workers who use drugs at especially high risk of police harassment, extortion, sexual coercion, and arrest [51].

In multiple settings, rates of injecting drug use and problematic drug use have been found to be higher among incarcerated women than among their male counterparts [57]. In some settings, HIV prevalence is higher among women prisoners than among men [58]. Because of financial constraints and logistical or bureaucratic obstacles, however, programs sometimes prioritize male prisoners, operating only in men's prisons and leaving women without essential care [10, 12, 13]. For example, a 2008 survey of women's access to OST in prisons found that in the Republic of Georgia, methadone was available in some men's prisons but not in women's prisons [13]. Four years later this is still the case, with methadone detoxification available in two pretrial detention facilities for men, but in no women's facilities [59]. In Kyrgyzstan, though methadone programs were planned for women's prisons, funding cuts have meant that they are still unavailable; OST is available only in men's prisons [10]. Work is needed to ensure that all prisoners, regardless of sex, have uninterrupted access to necessary health services (including NSP, OST, and ART) while incarcerated, including during pretrial detention.

### 1.6. Housing and Other Aspects of Social Stability

A strong association has been established between unstable housing and HIV risk [60]. Homeless women may trade sex for shelter, a situation that can make them extremely vulnerable on many levels. In general, poverty can lead women to trade sex for drugs, food, or other necessities; in such situations, concerns about HIV can be less urgent than immediate survival [35, 60–62]. A recent study analyzing the effects of multiple dimensions of social instability—including housing, employment, and incarceration—on the HIV risk practices of low-income women in Baltimore, USA found that increased social stability was associated with decreased HIV risk practices related to sexual practices and drug use. Rather than acting incrementally and independently, the various dimensions of social stability were found to be “cumulatively and synergistically linked to HIV risk behavior” for the women studied. The study found that homelessness was the only indicator that was consistently associated with every one of the HIV-related outcomes, confirming that housing plays a crucial role in HIV risk for women [63].

Given the central role that social stability plays in the HIV risk practices of women drug users, an effective HIV prevention strategy for this group needs to address housing, employment, legal status, and other factors underpinning social stability. Women do not make decisions about safer sex or drug use in a void; their decisions are shaped by their living situations, relationships, and economic positions. An increasing number of researchers have found that health interventions are more effective when they take into account the broader context of women's behavior, rather than limiting themselves to the distribution of basic supplies and information.

## 2. Designing Health Services That Respond to the Needs of Women Who Inject Drugs

To date, there has been limited research on the efficacy of interventions specific to women who inject drugs [64]. This is partly because gender-sensitive services often mix multiple approaches, are tailored to the individual, and are relatively long-term interventions that strive to address HIV risk practices in the specific context of women's lives. Services that combine structural, biomedical, and behavioral interventions can be more difficult to evaluate through randomized controlled trials (RCTs) measuring HIV incidence [65]. Even simpler services, such as NSP, need to achieve considerable coverage before they can have a substantial impact on HIV incidence or prevalence [66]. In some cases, lack of evidence of impact may reflect external limitations such as a cap on the number of syringes provided daily, rather than a problem with the intervention design [65].

To date, HIV risk reduction interventions among women IDU have been more successful in reducing drug-related risks than unsafe sexual behaviors, likely because of structural factors, gender power imbalances within society that have a strong effect on sexual relationships, and women's sense of self-efficacy and independence [64, 67, 68]. This points to a need for interventions that increase women's self-efficacy and autonomy as well as their awareness of the importance of safer sex, and that address gender inequities and inequalities.

To date, several interventions designed for women IDU have shown evidence of success (for further examples see [69]).

- A woman-focused intervention in an inpatient detoxification program in St. Petersburg, Russia, found that in comparison with the control group (which received nutritional counseling), women receiving the HIV-focused intervention reported a lower frequency of partner intoxication during their last sex act and a lower average number of unprotected vaginal sex acts with their main IDU sexual partner. Both groups reported lower levels of injection frequency. The two-session intervention consisted of educational activities, skill-building demonstrations, guided practice, and roleplaying, covering topics including drug use and relationships, physical and sexual abuse, rape and violence prevention, ways of discussing and negotiating safer sex, and developing a personalized action plan to help women reduce alcohol and drug use and HIV risk and avoid sexual and physical violence [70].
- In Baltimore, USA, the JEWEL intervention combined HIV prevention education and skills building with economic enhancement to reduce HIV risk among drug-using women (IDU and non-IDU) who traded sex for drugs or money. The HIV component aimed to increase women's knowledge about HIV, STIs, and drugs, improve their risk reduction knowledge and skills, and enhance self-efficacy and negotiation and communication skills to support safer sex. The economic component taught women how to make and sell jewelry, giving women practical skills while aiming to increase their self-efficacy in relation to licit employment. Self-reports three months after the intervention showed significant reductions in the exchange of drugs or money for sex, the median number of sex trade partners per month, daily drug use and daily crack use, the amount of money spent on drugs daily, and injecting drug use. There was also a small increase in the percentage of women reporting that they never shared needles (from 86.7% to 93.7%). Income from the jewelry sale was associated with a reduction in the number of sex trade partners at followup. The study suggested that exposing women to the possibility of gaining legal employment supports positive behavior change [71].
- In 2005, Family Health International Bangladesh established drug treatment services especially for women, leading to increasing numbers of women accessing treatment. Because OST was not available, treatment consisted of clonidine-assisted detoxification followed by three months of inpatient or outpatient care and followup. Women received HIV risk reduction counseling and voluntary counseling and testing, screening and treatment of STIs, overdose prevention education, and information on Hepatitis B and C. Counseling services were based on cognitive behavioral therapy and client-centered approaches. The services were free of charge, targeting homeless women with a history of drug-related harms. They were provided by specially trained female staff members and included childcare, prenatal care, and vocational rehabilitation. Treatment for male drug-using partners was offered to reduce barriers to treatment and poor treatment outcomes. A study of the program found that participation in the program was significantly associated with correct use of condoms, use of condoms during the last sex act, HIV testing, and correct assessment of risk [72].
- One review analyzed studies of alcohol and drug treatment programs for women that included childcare, prenatal care, women-only programs, supplemental services and workshops that address women-focused topics, mental health programming, and comprehensive programming. These components were positively associated with better treatment outcomes, reduced mental health symptoms, improved birth outcomes, employment, improved self-reported health status, and HIV risk reduction. One randomized study of pregnant methadone clinic patients who received prenatal care, therapeutic child care during visits, and relapse prevention support found improved outcomes at delivery and a threefold increase in the number of prenatal visits [73].
- A qualitative metasynthesis of studies of US and Canadian integrated drug treatment programs for pregnant or parenting women and their children found that programs that combined medical and social support increased women's sense of self and personal agency, increased women's engagement with the program staff and sense of giving and receiving support, increased women's reported openness about feelings, improved women's ability to recognize patterns of destructive behaviors, and helped women set goals. These psychosocial processes were reported to play a role in women's recovery and contribute to favorable outcomes. The motivating presence of children during treatment was also found to support women in their recovery. Perceived outcomes of programs included improved maternal and child wellbeing and enhanced parenting capacity [74].

In addition to the interventions described above, organizations in many parts of the world have provided HIV prevention and other health and social services for women who inject drugs. While most of these services have not been yet the subject of formal research, reports from the programs suggest that they have been useful in increasing the number of women IDU accessing health services and in more effectively addressing women's health and social needs [11, 41, 75].

Specialized programs for women drug users can take a variety of forms, from the very simple to the more sophisticated. On one end of the spectrum are basic additions to standard harm reduction packages (e.g., women's hygiene supplies, female condoms, pregnancy tests, woman-specific information materials, and diapers/baby supplies). Programs may designate special times for women to visit a center, have a staff member available to watch children while their mothers receive counseling or other services, or open women-only support groups. They can work to ensure a gender balance in their staff, train staff on gender issues, and address gender-specific needs. Many programs have found it useful to establish relationships with “trusted” gynecologists and other specialists who are familiar with drug use issues and who provide women with supportive, nonjudgmental care. Where possible, it is also desirable to give primary care providers and women's clinic staff basic training on drug use and HIV, so that they can identify possible cases and offer friendly referrals to nonjudgmental care [76]. 

These kinds of additions may be the most realistic option in settings, where harm reduction funding is very limited.

In the middle of the spectrum are new services based on an existing service site. Examples include counseling to respond to intimate partner violence and other trauma; parenting classes and work with women's children; mobile NSP, OST, and basic medical services for women unable to visit fixed service sites; legal aid to help women resolve problems with documents, housing, and access to social benefits [11, 38, 67]; economic empowerment efforts [71]; the provision of sexual and reproductive healthcare, including PMTCT [11, 43, 75]. These added services may be beyond the means of some programs, but can go a long way toward addressing the range of factors—including relationships, long distances to service sites, and legal and financial problems—that affect HIV risk behaviors.

Multidisciplinary case management, which weaves many kinds of services together, can help patients navigate the often complicated and intimidating network of medical and social services [77]. Case management is a way of working with a woman in context, addressing the range of problems she faces rather than isolating a single issue, such as safe injecting or HIV prevention.

At the upper end of the spectrum are stand-alone facilities for women IDU, which are more often seen in upper-income countries (see, e.g., [77, 78]). These can include special facilities and programs for pregnant and parenting women with a history of substance abuse and separate inpatient drug treatment/rehabilitation facilities for women. One important service is short-term/transitional housing for homeless women and their children. In many countries, women's shelters are closed to women with a history of drug use, or even to women with HIV. Programs in places like St. Petersburg have reported the positive results of short-term “crisis” housing for women IDU and their children [5].

The strict rules applied to OST programs are problematic for many patients, but may pose special difficulties for women with small children. A long trip to and from an OST clinic every day, with very limited hours, may be an insurmountable barrier for someone responsible for caring for a family (and, in many cases, working a job as well). Where possible, it is desirable to make OST available in multiple settings (e.g., from neighborhood pharmacies), to have flexible clinic hours, and to allow take-home doses when possible. Clinical protocols on OST should be established for maternity hospitals and similar settings in order to avoid treatment interruptions.

Women who use drugs should always be involved in the design and implementation of these programs, to ensure that programs are effective, appropriate, and respectful of human rights [79, 80].

Health service provision to people who use drugs, including drug-using women, is heavily affected by policy. Punitive policing practices, criminalization of possession of drugs and drug paraphernalia, and policies that restrict NSPs and OST have been shown to exacerbate drug-related risks and harms and drive people who inject drugs away from prevention and care services [81–85]. For instance, policies penalizing drug use during pregnancy or while parenting discourage women from seeking needed care, including drug treatment [44, 45]. It is essential that accessible treatment services be supported by policies that encourage women to seek treatment, rather than threatening them with jail time. Because women who use drugs often have insufficient access to sexual and reproductive healthcare, it is important that governments provide low-cost, accessible, nonjudgmental sexual and reproductive health care for high-risk women, including women who use drugs. Overall, it is essential to recognize and challenge national and international laws, policies, and practices that create risky drug-using environments and contribute to drug-related harms, in order to ensure the maximum impact of effective interventions [86, 87].

## 3. Conclusion

There is a clear need for more systematic collection of data on women who inject drugs globally. However, evidence indicates that this group faces a heightened risk of HIV as well as other harms and special barriers in accessing health care. Existing research and the experience of providers implementing gender-sensitive harm reduction and drug treatment programs indicate the importance of a multidisciplinary approach that addresses HIV risk practices in the context of women's relationships and social status. Sexual and reproductive health services, as well as the special needs of women with small children or with a history of IPV or other trauma, should be better incorporated into harm reduction and drug treatment services. Women prisoners often have unequal access to healthcare, and this discrimination should be remedied. Interventions should be developed to better respond to the specific needs of women who inject drugs, and governments should take these needs into account and formulate policy accordingly.

*Note*. This paper was adapted from “Developing effective harm reduction services for women who inject drugs,” a chapter in Harm Reduction International's 2012 report “The Global State of Harm Reduction: Toward an integrated response.”

## Figures and Tables

**Box 1:**
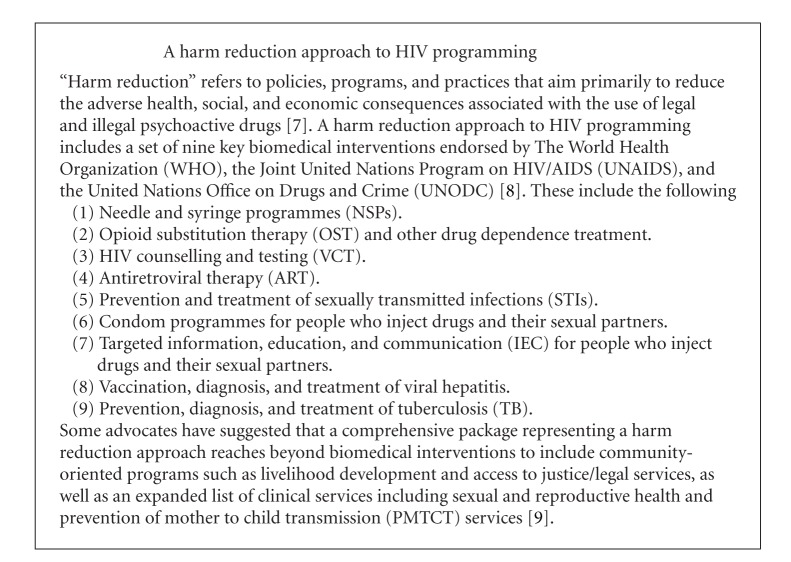


**Table 1:** Women as percentage of all IDU in selected countries.

Country/territory	Women as an estimated percentage of all IDU [unless otherwise noted, all estimates are from [4]]	Total estimated IDU population [all estimates are from [5]]	HIV prevalence among IDU (%) [all estimates are from [5]]
Cambodia	10%	1900	24.1
Canada	33% [6]	286,987	5.8
China	20%	2,350,000	6.4
Estonia	9% [3]	13,800	54.3–89.9
Georgia	10%	40,000	3.9
Indonesia	11%	105,784	36
Kenya	11%	49,167	18.3
Kyrgyzstan	10%	25,000	14.6
Malaysia	10%	170,000	8.7
Russian Federation	30%	1,815,000	37.15
South Africa	27%	67,000	19.4
Ukraine	26%	296,000	21.5
Vietnam	18%	158,414	13.4
